# Hepatitis B Immunoprophylaxis rarely prevents molecularly evident subclinical HBV infection in children born to HBsAg-positive mothers

**DOI:** 10.3389/fmicb.2026.1933288

**Published:** 2026-10-16

**Authors:** Liang Xu, Qinghai Dai, Huiying Yang, Rui Su, Yanli Shi, Jing Chen, Jing Hao, Jinyu Hao, Tomasz I. Michalak

**Affiliations:** 1 Department of Hepatology, Clinical School of the Second People's Hospital, Tianjin Medical University, Tianjin, China; 2 Department of Hepatology, Tianjin Second People’s Hospital, Tianjin, China; 3 Tianjin Institute of Hepatology, Tianjin, China; 4 Tianjin Integrated Traditional Chinese and Western Medicine Institute of Infectious Diseases, Tianjin, China; 5 Department of Obstetrics and Gynecology, Tianjin Second People’s Hospital, Tianjin, China; 6 Molecular Virology and Hepatology Research Group, Division of BioMedical Sciences, Faculty of Medicine, Health Science Center, Memorial University of Newfoundland, St. John’s, NL, Canada

**Keywords:** HBV, HBV immunoprophylaxis, HBV variant compartmentalization, maternal chronic HBV infection, subclinical HBV infection in newborns, mother-to-child HBV transmission

## Abstract

Hepatitis B virus (HBV) remains one of the most life-threatening human pathogens causing chronic hepatitis B and hepatocellular carcinoma in millions of people globally. Protective vaccines are available for decades, but their implementation is not yet universal. Mother-to-child transmission remains the prevailing route of HBV spread. Regardless of antiviral treatment of HBV-infected pregnant woman and neonatal immunoprophylaxis, HBV is transmitted at low levels to newborns as a subclinical (occult) infection at rates ranging from singular cases to the majority of the infant population examined in different centers worldwide. In the current study of 13 mother–child pairs, we applied a high-sensitivity molecular approach validated in previous studies, examination of different compartments of HBV occurrence, and testing of serial samples from infants followed for up to two years after birth to HBV-infected mothers treated or not with anti-HBV agent before parturition. All newborns received standard HBV immunoprophylaxis with HBV vaccine and hepatitis B immunoglobulin supplemented with additional doses of both during follow-up. The data showed that the majority (84.6%) of the children investigated remained HBV reactive during the 24-month follow-up after birth at levels of ≤100 HBV genome copies per microgram of DNA, indicating that the prophylactic protocol was only at a low level (~15%) effective in preventing subclinical occult infection in newborns. Nonetheless, the same protocol inhibited development of overt HBV infection for at least two years after birth. In addition, the sequencing of the 360-bp HBV preS-S genomic fragment revealed homology between infants and umbilical cord blood mononuclear cells that diverged from the corresponding sequences in maternal plasma or peripheral blood cells in 4 of the 5 mother–child pairs examined. These data from the single-center study indicate that subclinical HBV infection in infants born to HBV-infected mothers is far more frequent than that reported and argue that virus transmission to newborns could be an inevitable consequence of maternal infection with HBV. This study also uncovered previously unknown HBV genomic discrepancy between infected mothers and their infants with occult infection suggesting the fetal and/or placental origin of the viral variant detected.

## 1 Introduction

An estimated 254 million people have chronic hepatitis B virus (HBV) infection seropositive for HBV surface antigen (HBsAg) (World Health Organization, 2025). Although safe and effective vaccines are available for decades, HBV remains a serious global health problem with about 1.2 million of new symptomatic infections annually and up to 1.1 million deaths mainly due to liver failure, cirrhosis and hepatocellular carcinoma (HCC) (World Health Organization, 2025). In addition to the HBsAg-positive overt infection, persistent occult HBV infection (OBI) occurs with the expected rate of at least 10-fold greater than that of the overt infection. OBI is diagnosed when HBV DNA occurs in the absence of HBsAg detectable by the current clinical tests (Coffin et al., 2021; Michalak et al., 1994; Raimondo et al., 2019; Michalak, 2020). OBI is subdivided into seropositive and seronegative based on the presence or not of antibodies to HBV antigens, particularly those against HBV core (nucleocapsid) (anti-HBc) (Coffin et al., 2021; Mulrooney-Cousins and Michalak, 2015; Raimondo et al., 2019). The virological and pathogenic implications of OBI include risks of HBV transmission *via* blood transfusions and organ, tissue or cell donations, reactivation of hepatitis B due to immunomodulatory and cytotoxic therapies, acceleration of the progression of coinciding liver diseases, and HCC development (Candotti et al., 2019; Eilard et al., 2019; Mak et al., 2020; Saitta et al., 2022; Shi and Zheng, 2020).

The most frequent route of HBV spread in global population remains mother-to-child transmission (MTCT). Blocking of this passageway is considered to be the key in limiting the virus perinatal transmission and the likelihood of chronic hepatitis B (CHB) in infants born to infected women (World Health Organization, 2024). In this regard, both treatment with anti-HBV directly acting agents (DAA) of HBsAg-positive mothers and HBV vaccination (HepBvac) and passive immunization with hepatitis B immunoglobulin (HBIG) of infants within 12 h of birth, followed by HepBvac boosters at one and 6 months of age, are recommended (Liu et al., 2022; World Health Organization, 2024). However, OBI was reported in the newborns worldwide regardless of the maternal and perinatal HBV prophylaxis in ranges from 3.1 to 64% when tested by assays with varied HBV DNA detection sensitivity (Foaud et al., 2015; Li et al., 2023; Lu et al., 2016; Pande et al., 2013; Shahmoradi et al., 2012; Su et al., 2013; Zhou et al., 2017). The mechanism of vertically acquired OBI in the context of maternal anti-HBV therapy and neonatal HBV-specific prophylaxis remains unclear. It is also uncertain whether additional immunoprophylactic measures, i.e., HepBvac and/or HBIG administration beyond conventional schemes, reduce subclinical incidence of HBV infection in children.

The main aim of the study was to assess OBI occurrence in seemingly HBV-negative infants subjected to enhanced passive-active HBV immunoprophylaxis after birth to HBsAg-positive, chronically HBV-infected mothers treated or not with DAA before parturition using the high-sensitivity HBV detection approach validated in the previous studies on clinically silent infections with hepatotropic viruses. Combining polymerase chain reaction (PCR) with signal detection by nucleic acid hybridization (NAH) (i.e., PCR/NAH), detecting HBV genome with sensitivity below 10 virus genome copies per mL of serum or per one microgram of total DNA (Coffin et al., 2011a, 2011b; Michalak et al., 1994, 1999), the great majority (84.6%) of the children investigated were found HBV positive during the 24-month follow-up after birth, indicating that the immunoprophylaxis applied has limited effectiveness in preventing OBI, however it inhibited a clinically overt infection coinciding with hepatitis for at least 2 years after birth. In addition, sequencing of the HBV preS-S genomic fragments revealed HBV sequence homology between infants and umbilical cord blood mononuclear cells but divergence from maternal blood HBV sequences in 4 of the 5 mother–child pairs examined, suggesting that variants in newborns with OBI might be of fetal and/or placental origin.

## 2 Materials and methods

### 2.1 Mother-infant pairs

Thirteen pregnant women infected with HBV who attended the Antenatal Clinic at the Tianjin Second People’s Hospital (Tianjin, PR China) between October 2014 to July 2016 and their infants were investigated. The following mothers were excluded: (1) less than 18 years old and older than 45 years, (2) drinking >20 g of ethanol per day, (3) co-infected with another virus or having (4) co-existing infectious disease, (5) drug-induced liver injury, (6) autoimmune hepatitis; (7) decompensated liver disease or (8) liver cancer of any kind. The mothers were divided into 3 study groups. Group A (*n* = 4) included mothers whose serum HBV DNA load during pregnancy was consistently lower than 500 genome copies, also called virus genome equivalents (vge), per mL of serum or plasma and who did not receive anti-HBV DAA therapy. Mothers in Group B (*n* = 8) had serum HBV DNA levels initially ≥ 1.5 × 10^6^ vge/mL and were treated with telbivudine (LdT; 600 mg qd) from 24–28 weeks of pregnancy until delivery. In this group, HBV load declined to < 500 vge/mL in 4 mothers, as measured at one month before parturition, while continued to be > 1 × 10^3^ vge/mL in other 4 (Table 1). A mother with HBV DNA repeatedly > 1 × 10^6^ vge/mL and having 1.5 × 10^8^ vge/mL one month before parturition, who had refused anti-HBV therapy, was included in Group C (Table 1). All women were serum HBsAg positive throughout pregnancy and up to delivery.

**Table 1:** Selected markers of HBV infection in 13 mothers with chronic HBV infection treated or not with DAA prior to delivery and HBV-specific immunoprophylaxis and HBV DNA detection in their children.

Group	Mother-infant pair designation	Mothers				Infants					
		qHBV DNA (vge/mL)^a,b^	HBsAg^a,c^	HBeAg^a,c^	DAA therapy	Gender	Weight at birth (g)	Additional HepBvac (month)^d^	Additional HBIG (day)^e^	HBV DNA vge/mL^f^	Overall HBV DNA detection^g^
A	Pair 1	< 500	POS	NEG	No	M	4,250	18	14	NEG	NEG
	Pair 2	< 500	POS	NEG	No	M	4,250	8, 24	14	NEG	NEG
	Pair 3	< 500	POS	NEG	No	F	2,900	no	14	1.2 × 10^2^	POS
	Pair 4	< 500	POS	NEG	No	F	3,500	no	14	8.1 × 10^1^	POS
B	Pair 5	1.5 × 10^4^	POS	POS	Yes	M	3,550	12	14	1.1 × 10^2^	POS
	Pair 6	1.5 × 10^6^	POS	POS	Yes	M	3,200	no	14	nt	POS
	Pair 7	< 500	POS	POS	Yes	F	3,600	24	14	nt	POS
	Pair 8	< 500	POS	POS	Yes	F	3,500	12	14	1.8 × 10^2^	POS
	Pair 9	< 500	POS	POS	Yes	F	3,400	24	14	1.5 × 10^2^	POS
	Pair 10	< 500	POS	POS	Yes	F	3,300	8	14	9.7 × 10^2^	POS
	Pair 11	1.8 × 10^3^	POS	POS	Yes	F	3,200	12	14	nt	POS
	Pair 12	2 × 10^3^	POS	POS	Yes	M	3,500	8, 12	14, 210	3.6 × 10^2^	POS
C	Pair 13	1.5 × 10^8^	POS	POS	No	F	3,250	no	14	nt	POS

NEG, negative; POS, positive; M, male; F, female; g, grams; vge, virus genome equivalents; HepBvac, recombinant HBV vaccine; HBIG, hepatitis B immune globulin. ^a^Evaluated at approximately one month before parturition. ^b^HBV DNA determined in serum by clinical quantitative PCR (sensitivity 500 vge/mL). ^c^See Supplementary Table 2 for details. ^d^Times of HBV vaccine administration in addition to doses given routinely within 12 h and at one and 6 months after birth. ^e^Time of HBIG administration in addition to HBIG dose given at 12 h postpartum. ^f^Evaluated at 7 months of age. HBV DNA determined by PCR/NAH assay (sensitivity <10 vge/mL). ^g^Overall detection of HBV DNA during the 24-month follow-up after birth as assayed by PCR/NAH (sensitivity <10 vge/mL).

### 2.2 Postnatal hepatitis B immunoprophylaxis

All infants received 100 IU of HBIG (Chengdu Rongcheng Pharmaceuticals, Chengdu, PR China) intramuscularly *(i.m.)* within 12 h after birth. An additional dose of 100 IU was administered 14 days after birth or as indicated (Table 1). As well, recombinant HepBvac (GlaxoSmithKline Biologicals SA, Rixensart, Belgium) was given *i.m.* at 10 μg (0.5 mL) per injection within the first 12 h after birth and one and 6 months thereafter. If antibodies to HBsAg (anti-HBs) declined below 200 mIU/mL during follow-up, the vaccine was given at 8 months of age and/or at later time points (Table 1).

### 2.3 Samples collection

Blood samples were obtained from mothers’ peripheral blood about one month prior to parturition. Maternal peripheral blood plasma (MPBP) and maternal peripheral blood mononuclear cells (MPBC; commonly called peripheral blood mononuclear cells or PBMC), as well as umbilical cord blood plasma (UCBP) and umbilical cord blood mononuclear cells (UCBC) were collected at delivery. MPBC and UCBC were isolated from acid-citrate-dextrose (ACD)-treated blood by a Ficoll-HyPague density gradient centrifugation, as reported (Michalak et al., 1994; Skardasi et al., 2018), and their pellets stored at −80 °C. Blood samples from infants were obtained at 2 days of life and at 2, 7, 12 and 24 months of age. Notably, it was not feasible to secure samples at all times indicated above due to family unwillingness to subject children to needle punctures or issues with children’s transportation to hospital. Another constraint was the limited availability of sample material from children for HBV DNA assessment by PCR/NAH since testing by clinical assays was accepted to be a priority. Regarding blood samples from children collected in the absence of anticoagulant, sera accumulating above the blood clots and the clots containing both blood cells and serum leftovers, called as whole blood clot (WBC), were collected separately and stored at −80 °C. For the high-sensitivity HBV detection by PCR/NAH in infants, WBC were used only.

The complete list of abbreviations identifying the samples examined is as follows: MPBP or MP, maternal peripheral blood plasma or maternal plasma; MPBC or MC, maternal peripheral blood mononuclear cells or maternal cells; UCBP, umbilical cord blood plasma; UCBC or UC, umbilical cord blood mononuclear cells or umbilical cells; WBC02 or B02, whole blood clot from infant at 2 days of life; WBC2 or B2, whole blood clot from 2 months old infant; WBC7 or B7, whole blood clot from 7 months old infant; WBC12 or B12, whole blood clot from 12 months old infant, and WBC24 or B24, whole blood clot from 24 months old infant.

### 2.4 Evaluation of immunovirological markers of HBV infection

HBV infection markers were evaluated using the Roche Diagnostics GmbH system (Mannheim, Germany) and Cobas E601 electrochemiluminescence analyzer (Basel, Switzerland). Following the manufacturer’s instructions, HBsAg was considered negative when reading was < 0.05 IU/mL, anti-HBs negative when < 10 mIU/mL, HBV e antigen (HBeAg) negative when cut-off index (COI) was < 1.0, antibodies to HBeAg (anti-HBe) considered negative when COI ≥ 1.0, and anti-HBc negative when COI was > 1.0.

### 2.5 HBV DNA testing by clinical quantitative PCR assay

Total DNA was extracted from 200 μL serum or plasma of mothers and of their infants using the High Pure Viral Nuclei Acid kit (Roche Diagnostics, Branchburg, NJ, USA). HBV DNA load was determined by in-house quantitative PCR with HBV surface (S) gene primers using a LightCycler Roche LC480 instrument (Roche Diagnostics, Penzberg, Germany). The assay sensitivity was 500 vge/mL. Samples collected from children at the age of 2 years were tested by the same assay at the sensitivity of 100 vge/mL.

### 2.6 HBV DNA detection by high-sensitivity PCR/NAH assay

DNA was extracted from mothers’ MPBC and UCBMC and from infants’ WBC using a QIAamp DNA Blood Kit (Qiagen GmbH, Hilden, Germany) and from one mL of serum or plasma obtained from peripheral and cord blood of mothers. 2.5–3 μg DNA from MPBC, UCBMC and WBC, and DNA recovered from the whole volume of serum or plasma served as templates for direct PCR amplification and then 10 μL of the product was used for nested PCR. HBV S and X gene-specific primers were used in both runs under conditions reported previously (Coffin et al., 2011a, 2011b; Michalak et al., 1994) (Supplementary Table S1). Each amplification reaction was supplemented with positive amplification and negative contamination controls, and with ten-fold dilutions of complete recombinant HBV as quantitation standards, as reported (Coffin et al., 2011a; Michalak et al., 1994). To verify specificity of the amplified HBV signals and to augment the assay sensitivity, NAH was used (Coffin et al., 2011a, 2011b; Michalak et al., 1994). The sensitivity of nested PCR/NAH was 5–10 vge per mL or per one microgram of total DNA. This sensitivity was comparable to that of PCR/NAH assays designed to detect low levels of hepatitis virus genomes in other studies (Coffin and Michalak, 1999; Michalak et al., 1999; Mulrooney-Cousins et al., 2014; Pham et al., 2004; Pham et al., 2008; Skardasi et al., 2018; Gujar et al, 2013).

### 2.7 HBV sequencing and phylogenetic analysis

Either direct or clonal sequence analysis of the HBV preS-S region encompassing the 360-bp fragment spanning between nucleotides 3,124–364, as enumerated based on the HBV sequence with GenBank accession number LC057377, was done to determine HBV sequence variations between mothers, UCBC and their offspring. The amplicons were purified and directly sequenced or cloned using the TOPO-TA cloning system (Invitrogen, Carlsbad, CA, USA) and then sequenced, as reported (Chen et al., 2015; Pham et al., 2004; Pham et al., 2008). Thus, direct sequencing was done of PCR products obtained from 2 mothers (9/F and 12/F), their UCBC, and WBC samples from their children. Regarding clonal sequencing, up to 11 randomly selected clones derived from nested PCR products obtained from 3 mothers (8/F, 10/F and 11/F), UCBC, and WBC of their children who were followed for up to 12 months after birth were analyzed. The clones were sequenced in both directions using universal forward and reverse M13 primers by applying an Ion S5 Next-Generation Sequencing System (Thermo Fisher Scientific, Waltham, MA, USA). Analysis of the sequencing data was done with help of the CLC Genomic Workbench software (Qiagen). Neighbor-joining tree phylogenetic construction was based on the Tamura-Nei model and completed using the MEGAv7 software (accessed at www.magasoftware.net).

### 2.8 Statistical analysis

Statistical analyses were done using the Statistical Package for Social Science (SPSS) for Windows, version 21.0 (SPSS Inc., Chicago, IL, USA). Non-normal variables were expressed as median with minimum and maximum values and analyzed using Mann–Whitney U test. Spearman correlation analysis examined the relationship between infant age and HBV infection markers. Values of *p* < 0.05 were considered as statistically significant.

### 2.9 Sequence accession numbers

Sequences of the HBV preS-S genomic fragments identified in this study were deposited in GenBank and received accession numbers from PZ03023 to PZ03033.

## 3 Results

### 3.1 HBV infection and liver function in pregnant mothers

Testing for HBsAg approximately a month before delivery confirmed that all 13 mothers were antigen positive (Table 1 and Supplementary Table S2). One (7/F) also was anti-HBs reactive (Supplementary Table S2), 9 serum HBeAg-positive, although 8/9 were LdT treated (Group B; Table 1), while the remaining 4 untreated were HBeAg negative (Group A; Table 1) of them 3 were anti-HBe-positive (Supplementary Table S2). Eleven of 13 mothers tested for anti-HBc were reactive (Supplementary Table S2). HBV was detected at >1 × 10^3^ vge/mL in 4 of 8 mothers treated with LdT and in a mother belonging to Group C (Table 1). The remaining 8 women were HBV DNA-negative when tested by the clinical assay with the sensitivity of 500 vge/mL (Table 1 and Supplementary Table S2). However, 7 of them carried HBV at the time of parturition in MPBP and/or MPBC and in UCBC at levels <1.9 × 10^2^ vge/mL when examined by the PCR/NAH assay (see below).

Biochemical indicators of liver function were within normal ranges in all 13 mothers prior to delivery with the exception of alkaline phosphatase (ALP) in 3 mothers (Supplementary Table S2). The elevated ALP (normal limit of 135 U/L) could be due to fat-rich diet and/or the enzyme release during fetal bone development (Joshi et al., 2010). Also, when otherwise considered to be normal alanine aminotransferase (ALT) levels in mothers with the higher HBV loads (i.e., ALT median 27.0 U/L with inter-quartile range of 21.0–35.5 U/L; *n* = 5) were compared to those with the lower HBV loads (i.e., ALT median 18.0 U/L with inter-quartile range of 15.2–19.0 IU; *n* = 8), a statistically significant difference was found (*p* = 0.039). This may indicate that a mild liver necroinflammation endured in mothers with the higher HBV loads and imply that ALT >18 U/L might be considered elevated in HBV-infected pregnant females. This coincides with the notion that the upper normal ALT level for healthy females should be 18 U/L (Ruhl and Everhart, 2012).

### 3.2 HBV detection in maternal plasma and cells from peripheral and umbilical cord blood

We had a rare opportunity to compare HBV detection in MPBP *vs* UCBP and MPBC *vs* UCBC at the time of parturition by the high sensitivity testing. The overall rate of HBV detection in MPBP samples was 50% (6/12), and it was 25% (1/4) for mothers belonging to Group A (i.e., LdT-untreated) and 57.1% (4/7) in Group B (i.e., LdT-treated). For MPBC, the overall rate of HBV detection was 84.6% (11/13) and for mothers in Group A was 50% (2/4) and those in Group B 100% (8/8). MPBP and MPBC from LdT-untreated 13/F mother (Group C) were HBV DNA-positive. Further, the overall rate of HBV detection in UCBC was the same as in MPBC, i.e., 84.6% (11/13), and the rates for Group A and Group B were 50% (2/4) and 100% (8/8), respectively. Curiously, all UCBP samples were HBV DNA-negative. This was confirmed by repeated testing with primers specific to HBV S (surface), X, and C (core) genes (Supplementary Table S1). The reason is uncertain but could be related to the presence of a factor or factors co-isolating with DNA that inhibits PCR, as it was shown for some substances previously (Demeke and Jenkins, 2010; Pardoe and Michalak, 1995). Thus, maternal mononuclear cells from either peripheral or umbilical cord blood gave the greatest and identical rates of HBV detection (~85%), the rate was lower in maternal peripheral plasma (50%), while umbilical cord plasma was not suitable for HBV genome detection in this study. These data showed that circulating mononuclear cells, which include T cells, B cells, NK cells and monocytes (Corkum et al., 2015), are the most reliable material for detection of low levels of HBV and they are consistent with findings in the model HBV infection in woodchucks, hepatitis C virus (HCV) infection, and in a number of other infections with lymphotropic viruses (Coffin et al., 2021; Lee et al., 2006; Michalak, 2025; Michalak et al., 2004; Pham and Michalak, 2008).

### 3.3 Overall characteristics of HBV infection markers in infants receiving immunoprophylaxis

All children were serum HBsAg negative at 2 days and 2 and 7 months, 12 months (*n* = 12), and 24 months (*n* = 11) after birth (Table 2 and Supplementary Table S3). The rates of anti-HBs positivity were 15.4% (2/13) at 2 days postpartum, 100% (13/13) at 2 months of age, 92.3% (12/13) at 7 months, 100% (12/12) at 12 months, and 100% (11/11) at 24 months (Table 2). Two newborns at 2 days carried anti-HBs at levels <100 mIU/mL (Supplementary Table S3). HBeAg was detected in 53.8% (7/13) at 2 days and 15.4% (2/13) at 2 months, but was not during the remaining 24-month observation (Table 2). Anti-HBe were present in 46.1% (6/13) at 2 days, 30.8% (4/13) at 2 months, and 23.1% (3/13) at 7 months, but was not detected at 12 and 24 months of age (Table 2). Anti-HBc were positive in all 13 neonates at 2 days and 2 months. Their detection declined to 84.6% (11/13) at 7 months and further to 58.3% (7/12) at 12 months, and rebounded to 90.9% (10/11) at 24 months of age (Table 2). HBV DNA was not detected throughout the 24-month follow-up by the available clinical assay, but it was detected when the high sensitivity PCR/NAH was used (Table 2). Thus, HBV was identified in 71.4% (5/7) at 2 months, 77.8% (7/9) at 7 months, and in 60% (6/10) at 12 months of age. At 24 months, 3 children were tested and 2 of them were HBV DNA positive (Table 2). The estimated WHV DNA loads ranged from 8.1 × 10^1^ to 2.1 × 10^3^ vge/mL (median 1.35 × 10^2^ vge/mL with inter-quartile range of 1.1 × 10^2^–2.07 × 10^2^ vge/mL) (Supplementary Table S3). Only two (15.4%) infants, both born to DAA-untreated mothers from Group A, were HBV DNA negative throughout the entire 2-year follow-up (Table 2). The remaining (11/13; 84.6%) were HBV reactive at one or multiple time points during this period. The highest rate of HBV detection of 77.8% (7/9) was at the age of 7 months (Table 2). Considering the total number of WBC samples examined, 68.9% (20/29) were HBV DNA positive by the high sensitivity testing. In addition, a correlation analysis assessing association between the infants age and HBV infection markers showed no significant correlation of age with anti-HBs (*p* = 0.297) or HBeAg (*p* = 0.757) levels, but a positive correlation with anti-HBe (*p* = 0.008) and anti-HBc (*p* = 0.033) levels, while HBV DNA load (*p* = 0.011) was negatively correlated with age.

**Table 2:** Overall profiles of HBV infection markers in infants during the 24-month follow-up after birth to HBsAg-positive mothers.

HBV infection marker	Time of follow-up				
	2 days	2 months	7 months	12 months	24 months
HBsAg*^a^*	0/13 (0%)	0/13 (0%)	0/13 (0%)	0/12 (0%)	0/11 (0%)
Anti-HBs*^a^*	2/13 (15.4%)	13/13 (100%)	12/13 (92.3%)	12/12 (100%)	11/11 (100%)
HBeAg*^a^*	7/13 (53.8%)	2/13 (15.4%)	0/13 (0%)	0/12 (0%)	0/11 (0%)
Anti-HBe*^a^*	6/13 (46.1%)	4/13 (30.7%)	3/13 (23.1%)	0/12 (0%)	0/11 (0%)
Anti-HBc*^a^*	13/13 (100%)	13/13 (100%)	11/13 (84.6%)	7/12 (58.3%)	10/11 (90.9%)
HBV DNA*^b^*	nt	5/7 (71.4%)	7/9 (77/8%)	6/10 (60%)	2/3 (66.7%)

nt, not tested. ^a^Evaluated by Cobas E601 electrochemiluminescence immunoassays (Roche Diagnostics, Basel, Switzerland). ^b^Evaluated by PCR/NAH assay (sensitivity <10 vge/mL).

### 3.4 Profiles of HBV infection in infants born to mothers with low or high HBV load before parturition

To recognize the potential impact of maternal infection on the HBV serological profile in their children, the occurrence of individual infection markers was reanalyzed taking into consideration maternal HBV load prior to delivery. Thus, the group with the maternal HBV loads < 500 vge/mL was designated as the Maternal Low Virus Load (MLVL) group and included infants: 1/M, 2 M, 3/F, 4/F, 7/M, 8/F, 9/F, and 10/F (*n* = 8), while the group with the maternal loads > 500 vge/mL was named as the Maternal High Virus Load (MHVL) group and included infants: 5/M, 6/F, 11/F, 12/M, and 13/M (*n* = 5) (Table 1 and Supplementary Table S3). As summarized in Table 3 and presented in detail in Supplementary Table S3, anti-HBs were detected at 2 days postpartum at levels < 100 mIU/mL in one newborn in each group that corresponded to rates of 12.5% (1/8) and 20% (1/5) for the MLVL and MHVL groups, respectively. From 2 months onwards, anti-HBs occurred at levels > 200 mIU/mL in almost all infants. The exception was 2/M from the MLVL group with anti-HBs < 200 mUL/mL at 2 months and negative at 7 months of age, although HBIG was given within 12 h and at 2 weeks after birth and HepBvac additional doses at 8 and 24 months of age (Table 1). At 7 months, anti-HBs also dropped < 100 mIU/mL in 10/F from the MLVL group and in 12/M from the MHVL group compared to the levels of > 300 mIU/mL at 2 months (Supplementary Table S3). This prompted injections of additional doses of HepBvac to both infants and 200 IU of HBIG to 12/M (Table 1). These interventions did not lead to any substantial increase in the anti-HBs level, which was slightly above 100 mIU/mL at 12 months in 10/F and remained < 100 mIU/mL at 12 and 24 months in 12/M (Supplementary Table S3). In addition, 7/M and 9/F from the MLVL group, and 11/F from the MHVL group were re-injected with HepBvac at 24 or 12 months, respectively, due to decreases in anti-HBs. Some infants with anti-HBs declining < 200 IU were not re-vaccinated because of temporal health issues such as elevated body temperature or diarrhea.

**Table 3:** Profiles of individual HBV infection markers over the 24-month follow-up of infants born to mothers with low or high HBV loads prior to parturition.

HBV infection marker	Mother subgroup	Time of follow-up				
		2 days	2 months	7 months	12 months	24 months
Anti-HBs^a^	MLVL	1/8 (12.5%)	8/8 (100%)	7/8 (87.5%)	7/7 (100%)	6/6 (100%)
	MHVL	1/5 (20%)	5/5 (100%)	5/5 (100%)	5/5 (100%)	5/5 (100%)
HBeAg^a^	MLVL	3/8 (37.5%)	0/8 (0%)	0/8 (0%)	0/7 (0%)	0/6 (0%)
	MHVL	4/5 (80%)	2/5 (40%)	0/5 (0%)	0/5 (0%)	0/5 (0%)
Anti-HBe^a^	MLVL	5/8 (62.5%)	4/8 (50%)	3/8 (37.5%)	0/7 (0%)	0/6 (0%)
	MHVL	1/5 (20%)	0/5 (0%)	0/5 (0%)	0/5 (0%)	0/5 (0%)
Anti-HBc^a^	MLVL	8/8 (100%)	8/8 (100%)	6/8 (75%)	5/7 (71.4%)	6/6 (100%)
	MHVL	5/5 (100%)	5/5 (100%)	5/5 (100%)	2/5 (40%)	4/5 (80%)
HBV DNA^b^	MLVL	nt	3/4 (75%)	5/7 (71.4%)	4/8 (50%)	0/1 (0%)
	MHVL	nt	2/3 (66.6%)	2/2 (100%)	2/2 (100%)	2/2 (100%)

MLVL, mothers with low HBV load (< 500 vge/mL) at about one month before parturition; MHVL, mothers with relatively high HBV load (> 500 vge/mL) at approximately one month before parturition; nt, not tested. ^a^Evaluated by Cobas E601 electrochemiluminescence immunoassay system (Roche Diagnostics, Basel, Switzerland). ^b^Evaluated by in PCR/NAH assay (sensitivity < 10 vge/mL).

The HBeAg reactivity was detected at day 2 in 37.5% (3/8) of the MLVL newborns, but in 80% (4/5) of these from the MHVL group, but this difference was not statistically different (*p* = 0.26) (Table 3 and Supplementary Table S3). HBeAg remained positive at 2 months in 40% (2/5) of the MHVL infants, but not those in the MLVL group, and the antigen was not detected onward in either group (Table 3). The profile of anti-HBe was generally inverse to that of HBeAg. Hence, anti-HBe were detected at day 2 in 62.5% (5/8) of the MLVL newborns and in just 1/5 (20%) from the MHVL group (Table 3 and Supplementary Table S3). While anti-HBe remained detectable at 2 months in 50% (4/8) and at 7 months in 37.5% (3/8) of infants from the MLVL group, they were absent in the MHVL infants at any other time of follow-up than 2 days (Table 3).

Anti-HBc circulated in all newborns at 2 days and 2 months after birth (Table 3 and Supplementary Table S3) and were present in all (5/5) infants from the MHVL group and in 75% (5/8) of those from the MLVL group at 7 months. At 12 months, anti-HBc positivity rates declined to 71.4% (5/7) in the MLVL children and to 40% (2/5) in the MHVL group. Then, anti-HBc rebounded and were detected at 24 months in all (6/6) infants from the MLVL group and in 80% (4/5) of these born to mothers with higher HBV loads before parturition (Table 3).

As already pointed out, all infants were seemingly negative for HBV DNA during the 2-year follow-up when tested by clinical laboratory assay. Testing by PCR/NAH revealed HBV DNA positivity at a higher frequency in samples collected from the MHVL infants than in those from the MLVL group, i.e., 88.9% (8/9) *vs.* 60% (12/20), but difference was not statistically significant (*p* = 0.2) (Table 3 and Supplementary Table S3).

Overall, infants born to mothers with relatively low or high HBV loads prior to parturition showed different profiles of HBV infection markers during the 2-year follow-up after birth. While HBsAg was uniformly undetectable, the HBeAg and anti-HBe profiles were the most distinct between infants belonging to MLVL or MHVL group. Anti-HBc tended to have biphasic profiles in both groups and their levels decreased between 7 to 12 months after birth and rebound at 24 months of age. This may suggest a decline in anti-HBc transferred from mothers and initiation of synthesis of own antibodies due to ongoing HBV infection. HBV DNA was detected in the majority of samples available for analysis by the high sensitivity testing. The virus greatest positivity rate was around 7 months of age in children born to mothers with the high HBV load prior parturition.

Furthermore, analysis of the association between HBV infection markers and the length of follow-up of children in the MLVL subgroup revealed that there was no significant correlations of their age with anti-HBs levels (*p* = 0.155) or HBV DNA loads (*p* = 0.125), whereas there was a positive correlation with HBeAg (*p* < 0.001), anti-HBe (*p* < 0.001), and anti-HBc (*p* = 0.002) levels. In the MHVL subgroup, the age did not correlate with anti-HBs (*p* = 0.297) or HBeAg (*p* = 0.757) levels, but positively correlated with anti-HBe (*p* = 0.008) and anti-HBc (*p* = 0.033) levels, and negatively with HBV DNA load (*p* = 0.011). These age- related associations were partially distinct in the MLVL and MHVL subgroups.

### 3.5 Profiles of HBV infection in infants born to HBsAg-positive mothers treated or not with DAA before parturition

To evaluate the effect of gestational DAA treatment on the HBV infection in newborns, the profiles of HBV infection immunovirological markers and HBV DNA were compared in infants born to the LdT-treated and the untreated mothers. Accordingly, the group of infants born to the treated mothers (Group B; Table 1) included 5/M, 6/F, 7/M, 8/F, 9/F, 10/F, 11/F, and 12/M (*n* = 8) and was designated as the Infant-From-Treated-Mother (IFTM) group. The Infant-From-Untreated-Mother (IFUTM) group included infants 1/M, 2 M, 3/F, 4/F, and 13/M (*n* = 5) (Groups A and C; Table 1) Since infants born to mothers who reached lower HBV load (i.e., < 500 vge/mL) due to LdT therapy (*n* = 4) and these born to the LdT-treated mothers in which the treatment was not successful in lowering HBV below 500 vge/mL (*n* = 4) were included in the IFTM group (Table 3), these two subgroups were also compared.

Considering anti-HBs, they were detected 2 days after birth in two newborns in the IFTM group (2/8; 25%) of which one belonged to the MLVL subgroup and another to the MHVL group, while none from the IFUTM group was positive (Table 4). Anti-HBs were negative during the remaining 2-year observation except 2/M at 7 months of age (Supplementary Table S3).

**Table 4:** Profiles of individual HBV infection markers over the 24-month follow-up of infants born to mothers treated or not with a DAA compound prior to parturition.

HBV infection marker	Infant subgroup	Time of follow-up				
		2 days	2 months	7 months	12 months	24 months
Anti-HBs^a^	IFTM	2/8 (25%)	8/8 (100%)	8/8 (100%)	7/7 (100%)	7/7 (100%)
	IFUTM	0/5 (0%)	5/5 (100%)	4/5 (80%)	5/5 (100%)	4/4 (100%)
HBeAg^a^	IFTM	6/8 (75%)	2/8 (25%)	0/8 (0%)	0/7 (0%)	0/6 (0%)
	IFUTM	1/5 (20%)	0/5 (0%)	0/5 (0%)	0/5 (0%)	0/5 (0%)
Anti-HBe^a^	IFTM	2/8 (25%)	0/8 (0%)	0/8 (0%)	0/7 (0%)	0/7 (0%)
	IFUTM	4/5 (80%)	4/5 (80%)	3/5 (60%)	0/5 (0%)	0/4 (0%)
Anti-HBc^a^	IFTM	8/8 (100%)	8/8 (100%)	6/8 (75%)	4/7 (57.1%)	7/7 (100%)
	IFUTM	5/5 (100%)	5/5 (100%)	5/5 (100%)	3/5 (60%)	3/4 (75%)
HBV DNA^b^	IFTM	nt	5/6 (83.3%)	5/5 (100%)	5/6 (83.3%)	1/2 (0%)
	IFUTM	nt	0/1 (0%)	2/2 (100%)	1/4 (25%)	1/1 (100%)

IFTM, infants born to DAA-treated mothers; IFUTM, infants born to untreated mothers; nt, not tested. ^a^Evaluated by Cobas E601 electrochemiluminescence immunoassay system (Roche Diagnostics, Basel, Switzerland). ^b^Evaluated by PCR/NAH assay (sensitivity < 10 vge/mL).

HBeAg was positive on day 2 in 75% (6/8) of the IFTM newborns, among whose 3 belonged to MLVL and 3 others to MHVL subgroup, and in 1/5 (20%) of the IFUTM newborns (Table 4 and Supplementary Table S3). The antigen was also detectable at 2 months in 25% (2/8) in the IFTM group and both infants belonged to the MLVL subgroup. After this point, all children became HBeAg-negative until the end of follow-up (Table 4). Anti-HBe showed opposing profiles to the HBeAg detection. Hence, while they were reactive at 2 days of life in 25% (2/8) of the IFTM newborns (one of each in MLVH and MHVL subgroups) and in 80% (4/5) of those in the IFUTM group, they were not detected at any later time points in the IFTM group. In contrast, anti-HBe remained detectable for up to 7 months in 3/5 (60%) in infants from the IFUTM group (Table 4 and Supplementary Table S3).

Anti-HBc were identified in all newborns at 2 days and 2 months of age from the IFTM (8/8) and IFUTM (5/5) groups (Table 4). At 7 months, 75% (6/8) in the IFTM group, among them 4 from the MHVL subgroup and 2 from the MLVL subgroup, and all (5/5) in the IFUTM group were reactive. Anti-HBc reached the lowest positivity rate at 12 months after birth in both IFTM (57.1%; 4/7) and IFUTM (60%; 3/5) groups and rebounded to 100% (7/7) and 75% (3/4) at 24 months, respectively (Table 4).

Regarding HBV DNA detection by PCR/NAH, 16/19 (84.2%) samples from the IFTM group and 4/8 (50%) from the IFUTM group collected during the 24-month follow-up were positive. Among the 16 positive samples from the IFTM infants, 56.2% (9/16) belonged to the MLVL and 43.7% (7/16) to the MHVL subgroup (*p* = 0.72). Considering the rate of HBV carriage among infants at 12 and/or 24 months of age, 2/2 infants tested from the IFUTM group and 6/6 from the IFTM group were HBV positive. Among 6 IFTM infants, 3 were from MLVL and 3 others from the MHVL subgroup (Supplementary Table S3). Taken together, the data indicated that the mothers LdT treatment did not influence OBI incidence in their children.

Overall, the HBeAg and anti-HBe patterns were most divergent over time between infants belonging to the IFTM or IFUTM groups. While the HBeAg and anti-HBe profiles in the IFTM group more closely corresponded to the those in the MHVL group, the profiles in the IFUTM group resembled those in the MLVL group (Tables 3, 4). It could also be noted that: (1) both IFUTM and MLVL groups were characterized by a very brief period of HBeAg positivity and that anti-HBe ceased after 7 months of age; (2) both IFTM and MHLV groups displayed HBeAg up to 2 months in about a half of the infants, while anti-HBe vanished shortly after birth and became undetectable from 2 months onwards, and (3) The data suggested that both HBeAg and anti-HBe were passively transmitted from mothers and neither HBeAg re-appeared nor anti-HBe were *de novo* produced in response to ongoing occult infection during the first 2 years of life.

### 3.6 Comparison of HBV PreS-S sequences derived from mothers, umbilical cord blood cells and infants

In an effort to assess potential differences in the HBV genomic sequences between mothers and their children, bidirectionally sequenced clones or directly sequenced amplicons of the 360-bp HBV preS-S genomic fragments derived from maternal plasma (MP) or maternal peripheral blood mononuclear cells (MC), umbilical cord blood mononuclear cells (UC), and whole blood of infants between 2 and 12 months of age (i.e., B2, B7 and B12) were comparatively analyzed. The HBV sequences identified in infants and UC were first compared to those from maternal MP and MC, and then those detected in children to those from UC.

Clonal sequencing of the preS-S amplicons from the mother-infant pair designated as Pair 8 showed essentially identical sequence in MP and MC compatible with HBV subgenotype C2 (reference GenBank AF539983) (Figure 1A), with the exception of two mismatched point mutations in MP and one in MC (Supplementary Figure S1A). However, the great majority of clones (18/25; 72%) derived from the child at 2, 7 and 12 months of age contained HBV C/D subgenotype (reference GenBank AY817515) (Figure 1A) and displayed multiple point mutations when compared to the maternal virus (Supplementary Figure S1A). These mutations represented 8.6% of the 360-bp sequence analyzed. Also, HBV sequences homologous with the C subgenotype (reference GenBank DQ089793) were detected in 7/25 (28%) from the infant at 2 and 12 months of age, respectively, but not in the clones from a sample collected at 7 months (Figure 1A and Supplementary Figure S1A). UC from Pair 8 were not available for analysis. The phylogenetic analysis indicated separate clustering of the HBV sequences in MP and MC from those identified in the child (Figure 1A).

**Figure 1:**
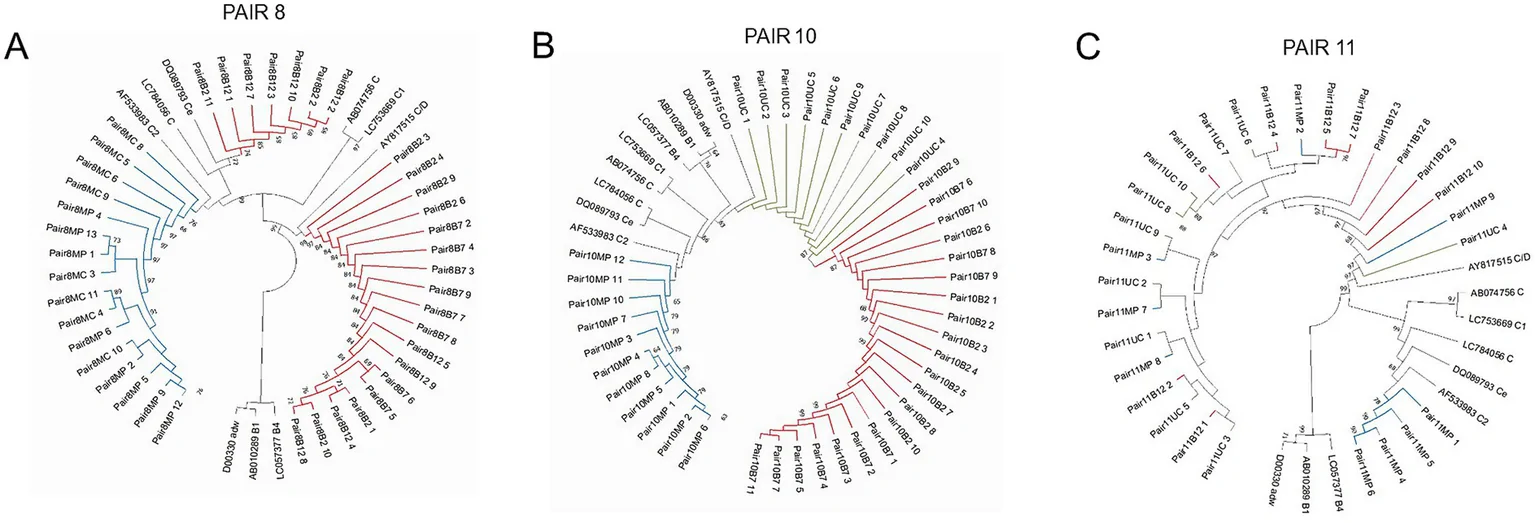
Neighbor-joining phylogenic tree of the cloned 360-bp fragments of the HBV preS-S genomic region derived from samples obtained from mother–child pair 8 **(A)**, pair 10 **(B)**, and pair 11 **(C)**. Samples identification: MP, mother plasma; MC, mother peripheral blood mononuclear cells; UC, umbilical cord blood mononuclear cells; B2, whole blood clot (WBC) containing blood cells and plasma from 2-month-old; B7, WBC from 7-month-old; and B12, from 12-month-old infant. Between 9 and 11 clones from individual samples are shown. Blue lines mark clones from MP and MC, green from UC, red from B2, B7 and B12, and black mark position of equivalent in the size and location nucleotide fragments from 9 GenBank sequences representing different HBV subgenotypes.

MP, UC, B2 and B7 samples were analyzed from Pair 10 and 10 to 11 clones per sample were sequenced and compared (Figure 1B and Supplementary Figure S1B). The data indicated that the preS-S sequences from the child at 2 and 7 months of age and from UC were identical, but they diverged from those in MP. This was a very compelling finding since all 10 of the 10 clones from UC and all clones from B2 and B7 samples displayed the same sequence homologous with HBV C/D subgenotype (Figure 1B). This variant diverged at 28 (7.8%) nucleotides from the 360-bp fragment identified in all 11 clones from MP, which carried C2 subgenotype (Figure 1B).

Likewise, MP, UC and B12 samples from Pair 11 were analyzed. Interestingly, MP displayed two HBV subgenotypes C2 and C/D (Figure 1C). They were found in 4/9 and 5/9 clones from MP, respectively, and differed at 27 (7.5%) nucleotides from each other (Supplementary Figures S1B,C). Clones obtained from 12-month-old infant and from UC showed the same sequence in all 10 clones examined that was identical with the maternal C/D sequence (Supplementary Figure S1B).

Direct sequencing of the preS-S amplicons was only feasible from the mother-infant pairs 9 and 12. The 360-bp fragments from MC, UC and B12 samples of Pair 9 and from MC, UC and B7 samples of Pair 12 were compared to the HBV genotype C reference sequences, and to each other within each pair (data not shown). Considering Pair 9, MC displayed HBV subgenotype C2. There were 21 single nucleotide mutations which were identical in UC and the infant B12 sample which were distinct from the HBV sequence in MC (data not shown). These point mutations constituted 5.8% of the 360-bp preS-S sequence carried in MC. Sequences in UC and B12 samples were closely matched the C/D subgenotype sequence. Overall, the HBV sequence fragment identified in the Pair 9 infant was identical with that in UC, similarly as it was uncovered for children from Pairs 10 and 11 (Supplementary Figure S1B). Regarding Pair 12, the preS-S fragments from MC and UC and the infant B7 sample showed identical HBV genotype C sequence (Supplementary Figure S1C).

These data showed the unexpected uniformity of a variant mutation within the HBV preS-S sequences in children and the umbilical cord blood mononuclear cells, which had clearly diverged from the equivalent maternal HBV sequence in 3 of 5 mother-infant pairs examined. In addition, the preS-S sequence in the Pair 8 child was identical with these found in children from Pairs 9, 10 and 11. Thus, among a total 32-point mutations in the preS-S sequence in the children, which differentiated from those in their mothers, 21 (65.5%) were shared by all four mother–child pairs. Four other single nucleotide point mutations were common for Pairs 8, 10 and 11 but not Pair 9, and two others for Pairs 8 and 10 but not occurred in Pairs 9 and 11. There were only 5/32 (15.6%) point mutations that were unique for the particular mother-infant pairs and they were found in Pairs 8, 10 and 11. Therefore, the results from 4 randomly selected pairs have shown surprisingly high degree of HBV variant homology between children and immune cells occurring in umbilical cord blood and, at the same time, a divergence from the equivalent maternal sequence. This may suggest a common mechanism causing the viral sequence dichotomy uncovered in this study.

## 4 Discussion

The current guidelines for HBV prevention of MTCT recommend HepBvac and HBIG administration to infants from HBsAg-positive mothers within 12 h after parturition followed by completion of the three-dose HepBvac series (World Health Organization, 2024). This passive-active prophylaxis has been shown to prevent clinically overt HBV infection in the majority of the newborns (Lee et al., 2006; Machaira et al., 2015; Sintusek et al., 2023), although it failed in 3% to almost 10% of the cases which developed with time serologically evident infection and CHB (Chen et al., 2012; Eilard et al., 2019; Lin et al., 2014; Pande et al., 2013). In contrast, anti-HBV DAA treatment of HBsAg-positive pregnant women in combination with the perinatal HBV immunoprophylaxis were unable, to a variable degree halt establishment of OBI in their children (Li et al., 2023; Lu et al., 2016; Pande et al., 2013; Zhou et al., 2017). To improve outcomes, HepBvac injections beyond the conventional scheme and delivery of additional doses of HBIG were attempted to augment blockage of HBV MTCT (Lin et al., 2014; Poovorawan et al., 1997; Sintusek et al., 2023). In the current study, the high-sensitivity molecular detection approach, which was previously successfully used to detect seronegative and seropositive HBV infections and subclinical infections with other hepatitis viruses (Chen et al., 2015; Coffin and Michalak, 1999; Michalak et al., 1994; Michalak et al., 1999; Michalak et al., 2004; Mulrooney-Cousins et al., 2014; Pham et al., 2004; Skardasi et al., 2018; Gujar et al., 2013), was employed to reassess OBI occurrence among newborns immediately after birth and after receiving enhanced HBV immune protective measures. The data showed that 84.6% of the children were HBV genome reactive during the 24-month follow-up after birth irrespectively of the type of immunoprophylaxis employed. Likewise, antiviral treatment of HBsAg-positive mothers in the last trimester of pregnancy did not inhibit HBV MTCT since all infants born to the LdT-treated mothers were found HBV DNA reactive at some point during follow-up. Collectively, the results showed that the preventive measures used had a limited effect on the OBI occurrence in newborns from HBsAg-positive mothers. The high rate of OBI incidence uncovered in this study was related to the high sensitivity of the HBV detection approach used, which included the assay of greater sensitivity, testing samples from different compartments of virus occurrence, and analysis of serial samples collected during the extended follow-up. Applying similar investigation strategy in another study, children born to 48 chronically HBsAg-positive mothers and subjected to the standard HBV MTCT preventive protocol, were followed for 12 months after birth, and their placentas and umbilical cord blood samples were examined (Mulrooney-Cousins, P.M. et al., unpublished data). At 12 months of age, 70.3% of the children were identified as HBV positive at loads around 1 × 10^2^ vge/mL of serum or plasma. In addition, 61.3% of the placentas examined were HBV DNA reactive and about 40% of them carried HBV genome replicative intermediates, such as virus RNA and/or virus covalently closed circular DNA (cccDNA). Regarding umbilical cord blood, HBV DNA was identified in 64% of the cases at similarly low levels as in children serum or plasma.

Furthermore, a study employing similarly sensitive virus detection approach was performed in the woodchuck model of hepatitis B in which dams with resolved acute hepatitis carrying traces of woodchuck hepatitis virus (WHV) (i.e., < 10^3^ vge/mL of serum) in the presence of otherwise protective anti-WHV envelope antibodies (anti-WHs, and equivalent of anti-HBs) universally transmitted WHV to their offspring (Coffin and Michalak, 1999; Michalak et al., 2004). Infection in newborns was serum WHV DNA positive, but negative for WHV surface antigen (WHsAg, an equivalent of HBsAg) and antibodies to WHV antigens, including antibodies to WHV core antigen (anti-WHc, an equivalent of anti-HBc), while WHV genome and molecular indicators of its replication were consistently detected in PBMC and organs of the immune system and, in some offspring, in livers (Coffin and Michalak, 1999; Michalak et al., 2004). This immunovirologically seronegative but molecularly evident WHV infection was designated as primary occult infection (POI) and extensively examined in the subsequent studies (Coffin et al., 2021; Gujar and Michalak, 2009; Gujar et al., 2013; Michalak et al., 2004; Mulrooney-Cousins et al., 2014). In contrast to POI, spontaneous or therapy-induced resolution of overt WHV infection with hepatitis is invariably followed by lifelong persistence of secondary occult infection (SOI). SOI is serum WHsAg-negative, but positive for WHV DNA and anti-WHc, while anti-WHs can be transiently detected during lifelong follow-up (Coffin et al., 2004; Michalak et al., 1999; reviewed in Michalak, 2025; Michalak, 2020). Notably, WHV in the course of both POI and SOI shows biophysical properties of complete virions and causes classical acute hepatitis and HCC after concentration by ultracentrifugation and administration to WHV-naive animals (Michalak et al., 1994; Michalak et al., 1999; Michalak et al., 2004; Mulrooney-Cousins et al., 2014). Because of strong molecular and pathogenic similarities between WHV and HBV (Chauhan et al., 2017; Michalak, 2025; Suresh and Menne, 2022), the immunovirological and molecular HBV infection profiles identified in children in our study indicate that the majority of them acquired seropositive OBI compatible with WHV SOI. However, this HBV DNA-positive, HBsAg-negative and anti-HBc-reactive occult infection became evident after clearance of maternal HBV-specific antibodies. This suggests that the newborns were exposed to relatively high HBV doses at some point during gestation, likely before initiation of anti-HBV LdT therapy in their mothers. However, it should be acknowledged that the prophylactic measures employed, although failed to block subclinical infection, suppressed the emerge of overt HBV infection for at least two years after birth.

Two of the infants born to DAA-untreated mothers, who carried low HBV loads during pregnancy, did not seemingly establish occult infection, as assumed from the repeatedly undetectable HBV DNA by the high-sensitivity approach used. However, both of them became anti-HBc reactive at the age of 2 years. This may suggest that they had seronegative OBI, an equivalent of WHV POI, but carried HBV at levels not detectable by otherwise very sensitive testing or they were *de novo* infected with HBV closely to the end of the observation period. In this regard, the detection of isolated anti-HBc (also called anti-HBc alone) in the absence of HBsAg and in the presence or temporal absence of detectable HBV DNA is recognized as an indicator of protracted low-level HBV infection (Coffin et al., 2004; Wang et al., 2017). Persistence of anti-HBc alone is considered to be indicative of repeated stimulation of the immune system by a trace synthesis of viral nucleocapsid. Experiments in the woodchuck model confirmed that isolated anti-WHc are indicative of SOI that can be accompanied by minimal to moderate hepatitis and may advance to HCC in some cases (Michalak et al., 1999; Michalak et al., 2004; Michalak, 2025). Since trans-placentally acquired maternal antibodies wane over the first 6 months of life, anti-HBc detected for the first time in the 2-year-old infants have to be produced in response to either *de novo* HBV infection or reactivation of ongoing seronegative POI. However, there also is a third possibility, the data from the woodchuck model informs that animals with POI remain susceptible to reinfection with WHV doses higher or equal to 10^3^ virions and in response produce anti-WHc, as well as reactivate WHV-specific T cell response (Gujar and Michalak, 2009; Mulrooney-Cousins et al., 2014; Gujar et al, 2013). Thus, at this stage, it remains unclear why exactly these two children became anti-HBc reactive so late after birth.

Despite a very low-level HBV infection in the children and in some of their mothers, the generation of HBV preS-S gene amplicons and their subsequent sequencing turned out to be feasible. The HBV preS-S fragment was selected for analysis because of its predisposition to mutations and forthright applicability for HBV genotyping. As the data showed, there was unexpected homology between the preS-S sequence found in the children and mononuclear cells of umbilical cord blood that diverged from the corresponding maternal sequences occurring in peripheral mononuclear cells and/or plasma. This was a compelling finding since it was encountered in 4 of the 5 mother–child pairs examined. This prompted search for the possible origin of such HBV genomic discrepancy based upon the data reported and our findings. In this regard, it has been shown that maternal blood mononuclear cells carrying HBV can cross the placental barrier, and facilitate virus intrauterine transmission and fetus infection (Bai et al., 2007; Lo et al., 1996; Shao et al., 2013; Shi et al., 2017; Wang et al., 2019; Xu et al., 2015). It also was established that PBMC infected with HBV, as well as with WHV, can support active virus replication and become a reservoir of the infectious and liver pathogenic virus (Coffin et al., 2011a; Coffin et al., 2021; Gao et al., 2017; Lu et al., 2009). Due to the hepadnaviral replication strategy, which engages the low fidelity transcription of virus pre-genomic RNA to DNA, HBV is highly prone to mutations resulting in multiple HBV subtypes, variants and in compartment sequence diversity (Glebe et al., 2021; Xie and Lu, 2024). Consequently, it could be expected that HBV replication in different milieus in a mother, fetus and/or placenta is prone to arise of distinct variants, including those potentially occurring within circulating immune cells. Since the traffic of these cells from the fetus to mother via the cord blood is possible (Lo et al., 1996; Lo et al., 2000), the uniformity of HBV variants in newborns and umbilical cord blood cells, as found in this study, is feasible. So far, a singular study has compared HBV diversity in sera between infants with OBI and their viremic mothers (Li et al., 2024). The analysis of the HBV whole sequences showed an increased complexity of point mutations in infants compared to their mothers. In this regard, since it is unlikely that the HBV sequence in our study diversified in children after birth and before the sample collection, this implies that such diversification occurred during gestation. However, another study examining HBV genetic diversity in maternal and umbilical cord blood plasma and placental samples did not find divergent variants in these three types of samples and suggested that mutations were unlikely raised due to intrauterine infection (Sirilert et al., 2023). Conversely, our examination of the preS-S sequences repeatedly identified almost uniform HBV variants in children and umbilical cord blood when cord blood mononuclear cells and whole blood cloths from children containing these cells were investigated. This indicates that circulating mononuclear cells, which include T and B lymphocytes, NK cells and monocytes (Corkum et al., 2015), should be examined when searching for origins of the HBV variants encountered in children born to HBV-infected mothers. In general, HBV variant uncovered in our study can be interpreted in support of two notions: (1) circulating lymphomononuclear cells can transmit HBV during fetal development and (2) either placenta or fetus or both could be the sites where these cells acquire variant HBV. More detailed investigations of the maternal PBMC may identify the variant matching that found in umbilical cord blood cells and children, but this will require a separate study. Regarding future study, the possibility that a given HBV variant may reside in particular immune cell subsets and may not be detectable in total PBMC should be taken into consideration, as it was uncovered in HCV infection (Michalak et al., 2007; Pham et al., 2008).

The possible placental origin of the HBV preS-S variant detected in this study cannot be excluded, however we did not have in hands suitable materials to confirm or discount this possibility. In this context, HBV infection of trophoblast cells and other cells in placenta has been reported (Wang et al., 2017; Xu et al., 2002; Zhao et al., 2022). Indirect evidence also come from infections with other viruses, including transmission of human immunodeficiency virus type 1 between infected T cells and placental trophoblast cells (Arias et al., 2003; Lagaye et al., 2001). Since HBV also is a lymphotropic virus (Coffin et al., 2021; Svicher et al., 2023), a similar route of viral spreading may operate within placentas of pregnant women infected with HBV.

By applying the high-sensitivity HBV detection approach, our findings document the much higher incidence of subclinical HBV infection in infants born to HBsAg-positive mothers than previously reported. The virus persisted for up to 2 years after birth, although all infants received enhanced HBV immunoprophylaxis. The treatment of mothers with HBV-specific DAA in the third trimester of pregnancy did not cease development of OBI. Taken together, our findings confirmed that the current approach to terminate HBV MTCT does not stop transmission of HBV traces capable of initiating subclinical infection in newborns. Infection of fetus early in pregnancy, prior to initiation of maternal anti-HBV therapy, might be one of the reasons why OBI was established. However, based on yet limited clinical data and considering findings in the woodchuck-WHV model of OBI, it appears that transmission of HBV traces throughout the placental barrier, fetus infection, and subsequent establishment of occult infection in children could be an inevitable consequence of maternal exposure to HBV. The long-term pathogenic outcomes of such acquired infection might not be clinically obvious or frequent, and recognition of their full extent will require much more effort using approaches strengthening sensitivity of HBV detection in the general population. Nonetheless, the documented ability of HBV and WHV to integrate into the host’s genome immediately after contact with susceptible cells and an indefinitely long persistence of their prooncogenic integrations may have serious life-treating consequences (Chauhan et al., 2017; Michalak, 2023; Michalak, 2025; Mulrooney-Cousins et al., 2014; Tu et al., 2017). Since there is currently not therapy able to completely eliminate HBV from the infected host, including treatment with tenofovir disoproxil fumarte (TDF) recommended to prevent vertical transmission in pregnant women (Terrault et al., 2018; EASL, 2025), our study emphasizes a tremendous importance of preventive vaccination of HBV-naïve infants and young adults to avert possibility of vertical virus spread in future. At the same time, search for more effective anti-HBV agents and more stringent MTCT preventive strategies has to continue to further reduce the transmission of HBV from already infected females.

## Data Availability

The datasets presented in this study can be found in online repositories. The names of the repository/repositories and accession number(s) can be found in the article/supplementary material.
