# Supplementary material for: Hepatitis B Immunoprophylaxis rarely prevents molecularly evident subclinical HBV infection in children born to HBsAg-positive mothers
Source: Front Microbiol. 2026 Oct 16;17:1933288. doi: 10.3389/fmicb.2026.1933288 (PMC13719598; doi:10.3389/fmicb.2026.1933288)
Supplement: Supplementary file 1 [file Supplementary_file_1.PDF]

Supplementary Table 1. Oligonucleotide primers used for HBV DNA amplification by PCR

Gene	Reaction	Primer	Location (nt)*	Sequence	Sense/Antisense
S	dPCR	SDP	2816-2838	5'-TTG TGG GTC ATA TTC TTG G-3'	sense
		SDM	450-472	5'-CGG GCA ACA TAC CTT GAT AGT CC-3'	antisense
	nPCR	SNP	3057-3078	5'-ATC CTC AGG CCA TGC AGT GG-3'	sense
		SNM	365-387	5'-GCA GAC ACA TCC AGC GAT AAC C-3'	antisense
X	dPCR	XDP	1248-1288	5'-CCA TAC TGC GGA ACT CCT AGC-3'	sense
		XDM	1781-1804	5'-ACA GAC CAA TTT ATG CCT ACA GCC-3'	antisense
	nPCR	XNP	1312-1331	5'-CTG GAG CAA ACA TTA TCG GG-3'	sense
		XNM	1750-1772	5'-CAA AGA CCT TTA ACC TGA TCT CC-3'	antisense
C	dPCR	CDP	1849-1871	5'-TGT TCA TGT CCC ACT GTT CAA GC-3'	sense
		CDM	2302-2321	5'-AAG ATA GGG GCA TTT GGT GG-3'	antisense
	nPCR	CNP	1895-1915	5'-TTT GGG GCA TGG ACA TTG ACC-3'	sense
		CNM	2276-2298	5'-ATA AGC TGG AGG AGT GCG AAT CC-3'	antisense

Abbreviations: nt, nucleotide number; dPCR, direct PCR; nPCR, nested PCR.

* Nucleotide positions according to the HBV DNA sequence under GenBank accession number LC057377.
