# Supplementary material for: Hepatitis B Immunoprophylaxis rarely prevents molecularly evident subclinical HBV infection in children born to HBsAg-positive mothers
Source: Front Microbiol. 2026 Oct 16;17:1933288. doi: 10.3389/fmicb.2026.1933288 (PMC13719598; doi:10.3389/fmicb.2026.1933288)
Supplement: Supplementary file 2 [file Supplementary_file_2.PDF]

Supplementary Table 2. Details on HBV infection markers and biochemical indicators of liver function in 13 chronically infected mothers at approximately one month before parturition.

Group	Mother designation	HBV infection markers						Liver biochemical indiactors								
		HBsAg (IU/mL)[a]	Anti-HBs (mIU/mL)[a]	HBeAg (COI)[a]	Anti-HBe (COI)[a]	Anti-HBc (COI)[a]	HBV DNA (vge/mL)[b]	ALT (U/L)[c]	AST (U/L)[c]	GGT (U/L)[c]	ALP (U/L)[c]	TBIL (μmol/L)[c]	DBIL (μmol/L)[c]	TP (g/L)[c]	ALB (g/L)[c]	A/G[c]
A	1/F	pos (2,978)	neg (<10)	neg (0.18)	nt	nt	neg (<500)	18	23	5	45	9.2	2.5	53.4	32.9	1
	2/F	pos (859)	neg (<10)	neg (0.15)	pos (0.04)	pos (0.008)	neg (<500)	18	18	19	200	9.5	2.4	66.8	35.8	1.2
	3/F	pos (2,937)	neg (<10)	neg (0.18)	pos (0.015)	pos (0.009)	neg (<500)	19	18	7	42	4.3	0.9	68.4	34.9	1
	4/F	pos (3,820)	neg (<10)	neg (0.18)	pos (0.005)	pos (0.009)	neg (<500)	15	15	42	105	8.1	1.9	67.5	35.4	1.1
B	5/F	pos (52,000)	neg (<10)	pos (1,756)	neg (9.64)	pos (0.012)	pos (1.5×10^4)	36	27	30	133	5	1.1	63	30.7	1
	6/F	pos (>52,000)	neg (<10)	pos (1,394)	neg (5.67)	pos (0.012)	pos (1.5×10^6)	24	22	7	129.5	12.9	3.8	58	35.4	1.6
	7/F	pos (12,196)	pos (95.5)	pos (8.28)	pos (0.48)	pos (0.01)	neg (<500)	30	44	18	332.9	15	5.3	63.6	33.4	1.1
	8/F	pos (24,471)	neg (<10)	pos (1,717)	neg (11.86)	pos (0.005)	neg (<500)	19	19	5	72	5.4	5.8	62	35	1.3
	9/F	pos (13,661)	neg (<10)	pos (1,244)	neg (5.34)	pos (0.009)	neg (<500)	16	22	6	77	9	4.1	64.7	36.2	1.3
	10/F	pos (42,231)	neg (<10)	pos (1,680)	neg (9.77)	pos (0.008)	neg (<500)	10	14	7	71	8.4	5.2	58.1	32.3	1.3
	11/F	pos (45,476)	neg (<10)	pos (1,838)	nt	nt	pos (1.8×10^3)	27	32	14	120	4.9	4.6	64.5	30.2	1.1
	12/F	pos (>52,000)	neg (<10)	pos (1,898)	neg (10.14)	pos (0.009)	pos (2×10^3)	35	40	14	128	5.8	5.8	60.3	33.6	1.3
C	13/F	pos (>52,000)	neg (<10)	pos (672.1)	neg (2.3)	pos (0.01)	pos (1.5×10^8)	18	21	7	110	11.1	4	66.2	33.6	1

Abbreviations: COI, cut-off index; vge, virus genome equivalents or virus copy number; ALT, alanine aminotransferase; AST, aspartate aminotransferase; GGT, gamma-glutamyl transpeptidase; ALP, alkaline phosphatase; TBIL, total bilirubin; DBIL, direct bilirubin; TP, total protein; ALB, albumin; A/G, albumin/globulin ratio.

[a] Evaluated by Cobas E601 electrochemiluminescence immunoassay system (Roche Diagnostics, Basel, Switzerland). HBsAg negative when < 0.05 IU/mL; anti-HBs negative when < 10 mIU/mL; HBeAg negative when COI < 1.0; anti-HBe negative when COI ≥ 1.0, and anti-HBc negative when COI ≥ 1.0.

[b] HBV DNA load determined by clinical quantitative PCR with sensitivity 500 vge/mL (see Participants and Methods).

[c] Evaluated by Hitachi 7180 automatic biochemical analyzer (Tokyo, Japan).
