# Supplementary material for: Hepatitis B Immunoprophylaxis rarely prevents molecularly evident subclinical HBV infection in children born to HBsAg-positive mothers
Source: Front Microbiol. 2026 Oct 16;17:1933288. doi: 10.3389/fmicb.2026.1933288 (PMC13719598; doi:10.3389/fmicb.2026.1933288)
Supplement: Supplementary file 3 [file Supplementary_file_3.PDF]

Supplementary Table 3. Immunovirological and molecular markers of HBV infection in HBIG-treated, HBV-vaccinated infants born to HBsAg-positive mothers at 2 days and 2, 7, 12 and 24 months of life

Infant number	2 days					
	HBsAg (IU/mL)[a]	Anti-HBs (mIU/mL)[a]	HBeAg (COI)[a]	Anti-HBe (COI)[a]	Anti-HBc (COI)[a]	HBV DNA (vge/mL)[b]
Infant 1/M	neg (<0.05)	neg (<10.0)	neg (0.06)	pos (0.007)	pos (0.012)	nt
Infant 2/M	neg (<0.05)	neg (<10.0)	neg (0.11)	pos (0.003)	pos (0.008)	nt
Infant 3/F	neg (<0.05)	neg (<10.0)	neg (0.11)	pos (0.019)	pos (0.006)	nt
Infant 4/F	neg (<0.05)	neg (<10.0)	neg (0.10)	pos (0.007)	pos (0.006)	nt
Infant 5/M	neg (<0.05)	neg (<10.0)	pos (236.9)	neg (2.03)	pos (0.008)	nt
Infant 6/M	neg (<0.05)	neg (<10.0)	pos (125.2)	neg (1.42)	pos (0.010)	nt
Infant 7/M	neg (<0.05)	neg (<10.0)	neg (0.07)	pos (0.281)	pos (0.013)	nt
Infant 8/F	neg (<0.05)	pos (20.3)	pos (164.0)	neg (1.9)	pos (0.008)	nt
Infant 9/F	neg (<0.05)	neg (<10.0)	pos (12.3)	neg (1.3)	pos (0.006)	nt
Infant 10/F	neg (<0.05)	neg (<10.0)	pos (146.7)	neg (1.67)	pos (0.007)	nt
Infant 11/F	neg (<0.05)	neg (<10.0)	neg (0.10)	pos (0.003)	pos (0.008)	nt
Infant 12/M	neg (<0.05)	pos (14.3)	pos (10.6)	neg (1.73)	pos (0.006)	nt
Infant 13/F	neg (<0.05)	neg (<10.0)	pos (76.9)	neg (1.197)	pos (0.010)	nt
Positive/ total tested	0/13	2/13	7/13	6/13	13/13	nt

Infant number	2 months					
	HBsAg (IU/mL)[a]	Anti-HBs (mIU/mL)[a]	HBeAg (COI)[a]	Anti-HBe (COI)[a]	Anti-HBc (COI)[a]	HBV DNA (vge/mL)[b]
Infant 1/M	neg (<0.05)	pos (272.1)	neg (0.14)	pos (0.025)	pos (0.008)	nt
Infant 2/M	neg (<0.05)	pos (159.6)	neg (0.11)	pos (0.003)	pos (0.010)	nt
Infant 3/F	neg (<0.05)	pos (316.2)	neg (0.11)	pos (0.061)	pos (0.007)	nt
Infant 4/F	neg (<0.05)	pos (358.8)	neg (0.11)	pos (0.044)	pos (0.007)	neg
Infant 5/M	neg (<0.05)	pos (302.8)	pos (5.72)	neg (1.34)	pos (0.009)	neg
Infant 6/M	neg (<0.05)	pos (364.2)	pos (3.55)	neg (1.44)	pos (0.020)	nt
Infant 7/M	neg (<0.05)	pos (299.5)	neg (0.08)	neg (1.08)	pos (0.017)	nt
Infant 8/F	neg (<0.05)	pos (477.8)	neg (0.21)	neg (1.41)	pos (0.010)	1.1×10^2
Infant 9/F	neg (<0.05)	pos (454.8)	neg (0.11)	neg (1.26)	pos (0.007)	1.1×10^2
Infant 10/F	neg (<0.05)	pos (324.0)	neg (0.14)	neg (1.44)	pos (0.007)	1.2×10^2
Infant 11/F	neg (<0.05)	pos (280.7)	neg (0.13)	neg (1.62)	pos (0.016)	2.1×10^2
Infant 12/M	neg (<0.05)	pos (322.1)	neg (0.12)	neg (1.62)	pos (0.007)	2.0×10^2
Infant 13/F	neg (<0.05)	pos (544.0)	neg (0.72)	neg (1.30)	pos (0.116)	nt
Positive/ total tested	0/13	13/13	2/13	4/13	13/13	5/7

Infant number	7 months					
	HBsAg (IU/mL)[a]	Anti-HBs (mU/mL)[a]	HBeAg (COI)[a]	Anti-HBe (COI)[a]	Anti-HBc (COI)[a]	HBV DNA (vge/mL)[b]
Infant 1/M	neg (<0.05)	pos (996.0)	neg (0.12)	neg (1.3)	pos (0.187)	neg
Infant 2/M	neg (<0.05)	neg (6.3)	neg (0.12)	pos (0.464)	pos (0.007)	neg
Infant 3/F	neg (<0.05)	pos (616.6)	neg (0.13)	pos (0.488)	pos (0.013)	1.2×10^2
Infant 4/F	neg (<0.05)	pos (>1000)	neg (0.12)	pos (0.88)	pos (0.005)	8.1×10^1
Infant 5/M	neg (<0.05)	pos (620.5)	neg (0.08)	neg (1.38)	pos (0.007)	1.1×10^2
Infant 6/M	neg (<0.05)	pos (>1000)	neg (0.33)	neg (1.52)	pos (0.048)	nt
Infant 7/M	neg (<0.05)	pos (667.4)	neg (0.24)	neg (1.59)	pos (0.023)	nt
Infant 8/F	neg (<0.05)	pos (392.2)	neg (0.12)	neg (1.62)	pos (0.025)	1.8×10^2
Infant 9/F	neg (<0.05)	pos (495.7)	neg (0.27)	neg (1.74)	neg (8.75)	1.5×10^2
Infant 10/F	neg (<0.05)	pos (18.81)	neg (0.27)	neg (1.77)	neg (7.81)	9.7×10^2
Infant 11/F	neg (<0.05)	pos (178.0)	neg (0.11)	neg (1.81)	pos (0.712)	nt
Infant 12/M	neg (<0.05)	pos (16.3)	neg (0.1)	neg (1.76)	pos (0.372)	3.6×10^2
Infant 13/F	neg (<0.05)	pos (>1000)	neg (0.08)	neg (1.03)	pos (0.027)	nt
Positive/ total tested	0/13	13/13	0/13	3/13	11/13	7/9

Infant number	12 months						24 moths					
	HBsAg (IU/mL)[a]	Anti-HBs (mIU/mL)[a]	HBeAg (COI)[a]	Anti-HBe (COI)[a]	Anti-HBc (COI)[a]	HBV DNA (vge/mL)[b]	HBsAg (IU/mL)[a]	Anti-HBs (mIU/mL)[a]	HBeAg (COI)[a]	Anti-HBe (COI)[a]	Anti-HBc (COI)[a]	HBV DNA (vge/mL)[b]
Infant 1/M	neg (<0.05)	pos (193.0)	neg (0.30)	neg (1.55)	pos (0.48)	neg	neg (<0.05)	pos (>1000)	neg (0.41)	neg (2.02)	pos (0.05)	nt
Infant 2/M	neg (<0.05)	pos (392.6)	neg (0.27)	neg (1.33)	neg (1.44)	neg	neg (<0.05)	pos (95.7)	neg (0.29)	neg (1.79	pos (0.04)	nt
Infant 3/F	neg (<0.05)	pos (448.5)	neg (0.24)	neg (1.37)	pos (0.39)	neg	neg (<0.05)	pos (413.4)	neg (0.32)	neg (1.73)	pos (0.10)	nt
Infant 4/F	neg (<0.05)	pos (358.8)	neg (0.11)	neg (0.04)	pos (0.007	1.9×10^{2}	nt	nt	nt	nt	nt	nt
Infant 5/M	neg (<0.05)	pos (187.2)	neg (0.29)	neg (1.15)	neg (6.57)	3.1×10^{2}	neg (<0.05)	pos (>1000)	neg (0.30)	neg (1.77)	pos (0.05)	nt
Infant 6/M	neg (<0.05)	pos (>1000)	neg (0.11)	neg (1.73)	neg (1.25)	neg	neg (<0.05)	pos (274.1)	neg (0.28)	neg (1.78)	pos (0.06)	7.9×10^{2}
Infant 7/M	neg (<0.05)	pos (331.5)	neg (0.11)	neg (1.72)	pos (0.67)	$2,1 \times 10^{3}$	neg (<0.05)	pos (83.1)	neg (0.27)	neg (1.74)	pos (0.06)	neg
Infant 8/F	neg (<0.05)	pos (33.8)	neg (0.29)	neg (1.82)	pos (0.75)	9.1×10^{2}	neg (<0.05)	pos (176.0)	neg (0.38)	neg (1.80)	pos (0.07)	nt
Infant 9/F	neg (<0.05)	pos (313.6)	neg (0.28)	neg (1.63)	neg (6.42)	2.9×10^{2}	neg (<0.05)	pos (179.9)	neg (0.43)	neg (1.96)	pos (0.07)	nt
Infant 10/F	nt	nt	nt	nt	nt	nt	nt	nt	nt	nt	nt	nt
Infant 11/F	neg (<0.05)	pos (45.7)	neg (0.24)	neg (2.01)	pos (0.07)	9.9×10^{2}	neg (<0.05)	pos (242.1)	neg (0.27)	neg (2.06)	pos (0.03)	nt
Infant 12/M	neg (<0.05)	pos (84.2)	neg (0.34)	neg (1.81)	pos (0.05)	nt	neg (<0.05)	pos (54.2)	neg (0.34)	neg (1.96)	pos (0.05)	nt
Infant 13/F	neg (<0.05)	pos (608.7)	neg (0.08)	neg (1.44)	neg (1.37)	nt	neg (<0.05)	pos (119.7)	neg (0.12)	neg (1.67)	neg (2.02)	9.2×10^{2}
Positive/ total tested	0/12	12/12	0/12	0/12	7/12	6/10	0/11	11/11	0/11	0/11	10/11	**2/3**

Abbreviations: M, male; F, female; neg, negative; pos, positive; COI, cut-off index; vge, virus genome equivalent or copy number; nt, not tested.

[a] Evaluated by Cobas E601 electrochemiluminescence immunoassay system (Roche Diagnostics, Basel, Switzerland). HBsAg, negative when < 0.05 IU/mL; anti-HBs, negative when < 10 mIU/mL; HBeAg, negative when < 1.0 COI ; anti-HBe, negative when ≥ 1.0 COI; and anti-HBc, negative when ≥ 1.0 COI.

[b] WHV DNA evaluated by research nPCR/NAH with sensitivity 5-10 vge/mL
