# Supplementary material for: Hepatitis B Immunoprophylaxis rarely prevents molecularly evident subclinical HBV infection in children born to HBsAg-positive mothers
Source: Front Microbiol. 2026 Oct 16;17:1933288. doi: 10.3389/fmicb.2026.1933288 (PMC13719598; doi:10.3389/fmicb.2026.1933288)
Supplement: Supplementary file 5 [file Supplementary_file_5.PDF]

## Mother-Child Pair 10

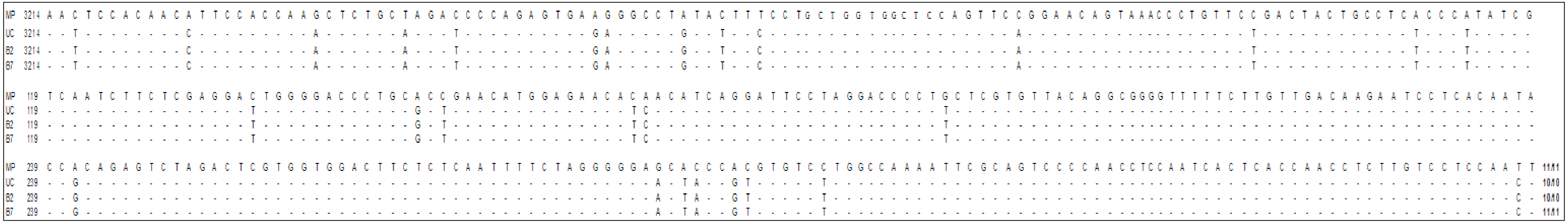

## Mother-Child Pair 11

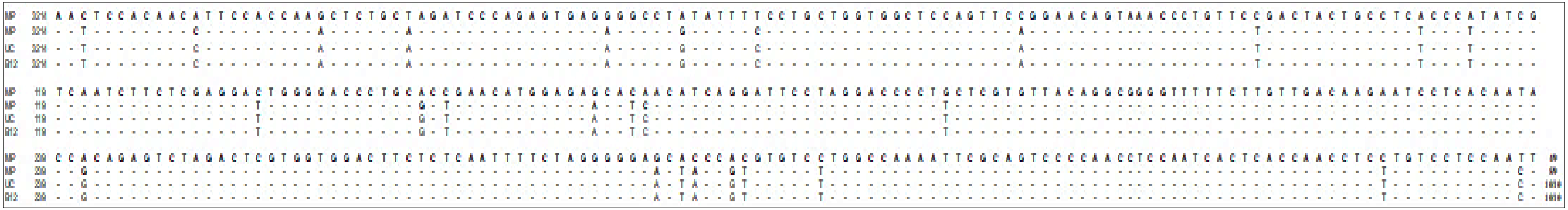

# Supplementary Figure 1B
